# Comprehensive molecular characterisation of epilepsy-associated glioneuronal tumours

**DOI:** 10.1007/s00401-017-1773-z

**Published:** 2017-10-20

**Authors:** Thomas J. Stone, Angus Keeley, Alex Virasami, William Harkness, Martin Tisdall, Elisa Izquierdo Delgado, Alice Gutteridge, Tony Brooks, Mark Kristiansen, Jane Chalker, Lisa Wilkhu, William Mifsud, John Apps, Maria Thom, Mike Hubank, Tim Forshew, J. Helen Cross, Darren Hargrave, Jonathan Ham, Thomas S. Jacques

**Affiliations:** 1 Developmental Biology and Cancer Programme, UCL Great Ormond Street Institute of Child Health, 30 Guilford Street, London, WC1N 1EH UK; 2 Developmental Neuroscience Programme, UCL Great Ormond Street Institute of Child Health, 30 Guilford Street, London, WC1N 1EH UK; 3 UCL Genomics, UCL Great Ormond Street Institute of Child Health, 30 Guilford Street, London, WC1N 1EH UK; 4 Department of Histopathology, Great Ormond Street Hospital for Children NHS Foundation Trust, Great Ormond Street, London, WC1N 3JH UK; 5 Department of Neurosurgery, Great Ormond Street Hospital for Children NHS Foundation Trust, Great Ormond Street, London, WC1N 3JH UK; 6 Department of Haematology and Oncology, Great Ormond Street Hospital for Children NHS Foundation Trust, Great Ormond Street, London, WC1N 3JH UK; 7 Department of Cellular and Molecular Diagnostics Service, Great Ormond Street Hospital for Children NHS Foundation Trust, Great Ormond Street, London, WC1N 3JH UK; 8 Department of Pathology, UCL Cancer Institute, London, WC1E 6DD UK; 9 Glioma Team, Division of Molecular Pathology and Cancer Therapeutics, The Institute of Cancer Research, London, SM2 5NG UK; 10 Centre for Molecular Pathology, Royal Marsden Hospital, London, SM2 5NG UK; 11 Department of Clinical and Experimental Epilepsy, UCL Institute of Neurology, London, WC1N 3BG UK

**Keywords:** Ganglioglioma, Dysembryoplastic neuroepithelial tumour, LEAT, Glioneuronal tumour, Epilepsy

## Abstract

Glioneuronal tumours are an important cause of treatment-resistant epilepsy. Subtypes of tumour are often poorly discriminated by histological features and may be difficult to diagnose due to a lack of robust diagnostic tools. This is illustrated by marked variability in the reported frequencies across different epilepsy surgical series. To address this, we used DNA methylation arrays and RNA sequencing to assay the methylation and expression profiles within a large cohort of glioneuronal tumours. By adopting a class discovery approach, we were able to identify two distinct groups of glioneuronal tumour, which only partially corresponded to the existing histological classification. Furthermore, by additional molecular analyses, we were able to identify pathogenic mutations in *BRAF* and *FGFR1*, specific to each group, in a high proportion of cases. Finally, by interrogating our expression data, we were able to show that each molecular group possessed expression phenotypes suggesting different cellular differentiation: astrocytic in one group and oligodendroglial in the second. Informed by this, we were able to identify CCND1, CSPG4, and PDGFRA as immunohistochemical targets which could distinguish between molecular groups. Our data suggest that the current histological classification of glioneuronal tumours does not adequately represent their underlying biology. Instead, we show that there are two molecular groups within glioneuronal tumours. The first of these displays astrocytic differentiation and is driven by *BRAF* mutations, while the second displays oligodendroglial differentiation and is driven by *FGFR1* mutations.

**Electronic supplementary material:**

The online version of this article (doi:10.1007/s00401-017-1773-z) contains supplementary material, which is available to authorized users.

## Introduction

Epilepsy is the most common serious chronic neurological condition of childhood, which has long-term effects on health and quality of life. In the UK, the incidence of epilepsy in children between 0 and 7 years of age has been estimated at 71-116/100,000 persons-years-at-risk [12]. In a separate study, the cumulative incidence of epilepsy rose to 8.4/1000 by the age of 23 [9]. Structural abnormalities of the brain are frequently observed in patients with intractable childhood epilepsy [1]. Of these abnormalities, brain tumours represent the second most common cause of seizures [5]. This is particularly true for low-grade cortical glioneuronal tumours, the most prevalent of which are ganglioglioma (GG) and dysembryoplastic neuroepithelial tumour (DNET).

Glioneuronal tumours pose a significant diagnostic problem. A number of subtypes are recognised on histological grounds, but in clinical practice are often poorly discriminated between by their histological features. Moreover, a high proportion of cases possess non-specific histological appearances that preclude confident classification. Poor inter-observer correlation is commonplace, particularly for ganglioglioma and DNET, where there is unexplained and marked geographical variability across surgical series, which suggests marked variability in diagnostic practice (Online Resource 1, reviewed in [16]).

It is unclear how many distinct entities are present within the spectrum of glioneuronal tumours, and a number of subtypes have been proposed [3]. However, these classifications are based on a subjective assessment of histological features that may not be representative of underlying tumour biology. Robust biologically informed classifications are necessary to facilitate effective characterisations of tumour subtypes.

A number of the previous studies have identified pathogenic abnormalities in a proportion of glioneuronal tumours. The most notable of these are the presence of *BRAF*-V600E mutations in a subset of ganglioglioma and *FGFR1* abnormalities in a portion of DNETs [13, 14]. However, *BRAF*-V600E mutations and copy number changes have also been reported in DNETs, and there is a general lack of consensus regarding the genetic background of these tumours [4, 7, 10]. A common theme among the previous studies is that they recruited and segregated cases according to classical histological features. However, as mentioned previously, this does not necessarily reflect the underlying biology and may be prone to inaccuracies in classification. In light of this, we hypothesised that a molecular classification, based on biological similarity, may align more closely with pathogenic features.

To approach this problem, we have used molecular techniques to characterise the most prominent glioneuronal tumours, including those with non-specific histology. We have taken a class discovery approach, to classify tumours according to their underlying molecular profiles and features. From this, we have been able to identify two distinct molecular groups within glioneuronal tumours. Moreover, we have been able to identify genetic abnormalities that are strongly enriched within each of the molecular groups unveiled. Finally, we have determined cell-type specific phenotypes that define each group. Here, we present a comprehensive genomic classification and characterisation of epilepsy-associated glioneuronal tumours, which we favour over the conventional histological approaches.

## Materials and methods

### Case recruitment

Surgical cases were principally retrieved from the Great Ormond Street Hospital (GOSH) archives. 111 patients diagnosed with a ganglioglioma, DNET, or glioneuronal tumour between 1991 and 2015 were identified and corresponding diagnostic formalin fixed paraffin embedded (FFPE) sections were retrieved. The histology for these cases was reviewed by an experienced paediatric neuropathologist (TSJ), according to current WHO diagnostic criteria [11]. Cases were categorised into three groups according to their histological features: Ganglioglioma, DNET, and glioneuronal tumour of uncertain histologic subtype (GNT NOS). The latter group included non-specific histological variants, atypical tumours, and those that appeared as a mix of histological types.

Additional cases were acquired from the Children’s Cancer and Leukaemia Group (*n* = 24) (CCLG Project Number 2015BS03). These cases were categorised histologically in the same manner as our GOSH cohort. Detailed histological review was not performed for these cases due to limited diagnostic slides.

For control cases in this study, we retrieved archival temporal cortex from patients that had undergone resections for hippocampal sclerosis (HS). Prior to inclusion, this material was assessed and confirmed to be free of tumour and other structural pathology.

### Clinical review

Clinical data were available for 69 patients within our GOSH cohort, including follow-up data for 60 of these patients that had presented with epilepsy. Data were acquired from archived patient records and included sex, age at first seizure, seizure duration prior to surgery, age at surgery, presence of tumour on postoperative MRI, and seizure status at 1 year postoperatively and last follow-up. Statistical analyses for associations with seizure outcome were performed using SPSS Version 24.

### RNA preparation and sequencing

RNA was extracted from frozen archival material from cases for which it was available. Prior to extraction, haematoxylin and eosin-stained cryosections were prepared to assess tumour content. Tumour content was graded 1–4 as follows: 1—no evidence of tumour; 2—focal areas of tumour; 3—majority is tumour but with some non-neoplastic components; and 4—extensive tumour/tumour comprised entire section. Cases where the cryosection was graded 1–2 were excluded. Extraction of total RNA was carried out using the miRNeasy Mini Kit according to the manufacturer’s instructions. Eluted RNA was subjected to quality control by analysis on a NanoDrop 1000 spectrophotometer and samples with a 260/280 ratio lower than 1.8 were excluded. Further quality control was carried out using the Agilent Bioanalyzer platform and samples with an RNA Integrity Number (RIN) lower than 4 were excluded. Library preparation and RNA sequencing were carried out by UCL Genomics. Libraries were prepared using non-strand specific Illumina TruSeq Sample Preparation Kits. Libraries were sequenced at a depth of 15 million reads per sample on the Illumina NextSeq 500 platform. Resultant FASTQ files were aligned using TopHat and Cufflinks software packages to produce Binary Alignment Map (BAM) files for subsequent analysis.

### Analysis of expression data

Bioinformatic analysis of expression data was primarily carried out in R. Read counts were generated from BAM files using the GenomicRanges package. Consensus clustering was carried out using the ConsensusClusterPlus package according to the Ward method for hierarchical clustering. Cases were clustered using the top 5000 most variable genes across the cohort (tumours and controls) as determined by median absolute deviation. Prior to analysis, cases were clustered in an unsupervised manner into two groups and those that clustered with controls were excluded as likely possessing low tumour content. In subsequent clustering, to identify the optimum number of clusters, the *k* corresponding to the first downwards inflection in cumulative distribution function was used (Online Resource 2). Identification of differentially expressed genes was performed using DESeq2. For differential expression analysis, a false discovery adjusted *p* value (*q* value) less than 0.1 was considered significant.

Gene set enrichment analysis (GSEA) was carried out using the GSEA software [15] (available at http://software.broadinstitute.org/gsea/index.jsp). Genes were pre-ranked in DESeq2 by Wald statistic before GSEA analysis against custom gene sets for neural-cell-type specific expression patterns. Neural-cell-type specific gene sets were constructed from the top 150 genes up-regulated in each cell type as reported by Zhang et al. [19].

### DNA preparation

DNA was extracted from FFPE tissue using the Maxwell 16 FFPE Tissue LEV DNA Purification Kit in conjunction with the Maxwell 16 Research Instrument according to the manufacturer’s instructions. Subsequently, 250 ng eluted DNA was subjected to bisulphite conversion, while the remainder was stored at − 80 °C for TAm-seq assay. Bisulphite conversion was performed using the Zymo EZ DNA Methylation-Gold Kit. Bisulphite converted DNA was additionally treated using the Infinium FFPE DNA Restore Kit.

DNA for target capture panel sequencing was extracted using the QIAamp DNA FFPE Tissue Kit. DNA was quantified using Qubit dsDNA High Sensitivity Assay Kit with the Qubit 2.0 fluorometer. Subsequently, analysis by TapeStation 2200 using the genomic DNA ScreenTape assay was performed to determine the degree of fragmentation.

### 450k methylation analysis

Bisulphite converted and restored DNA was assayed on the Illumina HumanMethylation450 BeadChip array platform, in accordance with the Infinium HD Assay protocol. Processed arrays were scanned using an Illumina IScan array scanner to generate IDAT output files.

Bioinformatic analysis of methylation data was performed in R. Data was read in using Minfi, and normalised with the included subset-quantile within array normalisation method. Probes located on the X and Y chromosomes were excluded. In addition, probes located within 50 bp of an SNP, probes known to cross-hybridise, probes with a minor allele frequency > 5%, and probes that had failed to hybridise in > 30% of samples in the cohort were excluded.

Consensus clustering of methylation data was carried out using the ConsensusClusterPlus package according the Ward method. Cases were clustered using the top 10,000 most variable CpGs across the cohort as determined by median absolute deviation. To identify the optimum number of clusters, *k* corresponding to the first downwards inflection in cumulative distribution function was used (Online Resource 2).

Copy number analysis on 450k methylation data was carried out using the conumee R package. LogR ratio thresholds of ± 0.15 were used as a cutoff to determine gains and losses.

Gene set enrichment analysis of methylation data was carried out using the *gsameth* function in the missMethyl R package. Differentially methylated positions (DMPs) between Group 1 and Group 2 tumours were identified using the *dmpFinder* function in Minfi. This list of DMPs was then assayed for over-representation of CpGs associated with genes specified by each gene set. Gene sets were constructed from the top 150 genes up-regulated in astrocytes and oligodendrocyte precursors, as reported by Zhang et al. [19].

### TAm-seq

Primers specifically targeting *CTNNB1* Exon 3*, BRAF*-V600, *HIST1H3B*, *H3F3A*, *IDH1,* and *IDH2* were designed. Sequencing was performed using a protocol adapted from Weaver et al. [18]. After two separate rounds of PCR, resulting amplicons consisted of the genomic region of interest flanked by adapter sequences, a 5′ sample-specific barcode, and Illumina adapter sequences complementary to the flow cell. Samples were pooled, purified, and sequenced on the Illumina MiSeq platform. Reads were aligned to the human genome using bwa mem (v0.7.13-r1126) and variants were detected by VarScan mpileup2snp (v2.3) with a minimum variant allele frequency of 0.5%. Regions were failed if fewer than 100 reads were observed using bamreadcount. Variants were considered valid if present in either of duplicate sequencing reactions with at least 50 reads in the variant allele.

### Capture panel sequencing

A panel covering a total of 78 genes, either recurrently altered in paediatric cancers or clinically actionable in adult cancers was used [6]. Customised biotinylated probes (Nimblegen SeqCap EZ library) capture a total of 311 kb for the detection of single nucleotide variants, short indels, copy number variants, and structural rearrangements. Libraries were prepared using the KAPA Hyper kit and SeqCap EZ adapters. Following fragmentation, DNA was end-repaired, A-tailed and ligated with indexed adapters. DNA was amplified over 6 PCR cycles where 200 ng starting material was available. 10 PCR cycles were performed where less than 200 ng was used. Samples were multiplexed and hybridised twice overnight on consecutive days using 1 µg of total pre-capture library DNA to the DNA baits targeting the 78 panel genes. 5 PCR cycles were performed between hybridisations to enrich the captured product. After the second hybridisation, unbound capture baits were washed away and the remaining hybridised DNA was amplified over 12 PCR cycles. PCR products were purified using AMPure beads and quantified using the KAPA Quantification Q-PCR Kit. Sequencing was performed using the Illumina MiSeq platform (75 bp paired-end reads and v3 chemistry) according to the manufacturer’s instructions.

Primary analysis was performed using the MiSeq Reporter Software (v2.5.1), generating nucleotide sequences and base quality scores in FASTQ format. Resulting sequences were aligned against the human reference genome build GRCh37/hg19 to generate binary alignment maps and variant call files. Secondary analysis was performed using an in-house Molecular Diagnostics Information Management System to generate QC, variant annotation, visualise data, and generate reports. All potential mutations, structural variants, and CNVs were visualised using the Integrative Genomics Viewer. In the absence of germline DNA, commercial genomic DNA G147A (Promega) was used as a baseline control to filter out common SNPs. In addition, all variants identified were screened against Exome Aggregation Consortium (ExAC) and dbSNP databases, and were excluded if their variant allele frequency across the population was > 0.0001 with 0 homozygous calls.

### Immunohistochemistry

Immunohistochemistry was performed using a Leica Bond-Max autostainer according to manufacturer’s protocol F. Cases were stained for CCND1 (1/40, CellMarque–241R), CSPG4 (1/100, Atlas–HPA002951) and PDGFRA (1/200, Santa Cruz–sc-338). Immunohistochemistry against H3 K27M (1:1500, Millipore – ABE419) was performed in the same manner. Positive controls for all antibodies were included (CCND1-Mantle cell lymphoma; CSPG4-Large intestine, PDGFRA-Gastrointestinal stromal tumour; H3 K27M-Diffuse midline glioma, H3 K27M-mutant). Staining for CCND1 and PDGFRA was undertaken at UCL Advanced Diagnostics.

## Results

### Clinical and histopathological features of the cohort

From a cohort of 111 archival cases retrieved from the GOSH archives, we identified 99 glioneuronal tumours upon histological review. The 12 remaining cases represented non-glioneuronal tumours that had been flagged during initial cohort collection due to the presence of the term “glioneuronal” in the comments of their archival pathology reports. The most common histological subtypes were ganglioglioma (44/99–44%), followed by DNET (18/99–18%). However, the remaining 37/99 (37%) of cases could not be definitively categorised due to a lack of distinctive histological features and were classified as “glioneuronal tumours of uncertain histologic type” (GNT NOS). Summary data for the histological features of the GOSH cohort, excluding 1 case with extremely limited material, are shown in Table 1. We obtained 24 additional tumours from the CCLG. 20 of these represented cortical glioneuronal tumours, comprising 16 ganglioglioma, 1 DNET, and 3 GNT NOS. The remaining four cases could not be confidently classified, due to extremely limited diagnostic material, and were excluded.

**Table 1:** Summary of histological features for 98 glioneuronal tumours

Histological features	Ganglioglioma (*n* = 43)	DNET (*n* = 18)	GNT NOS (*n* = 37)
Specific glioneuronal element	0% (0/43)	89% (16/18)	0% (0/37)
Floating neurons	2% (1/43)	72% (13/18)	11% (4/37)
Dysplastic neurons	100% (43/43)	0% (0/18)	13% (5/37)
Anaplasia	5% (2/43)	5% (1/18)	0% (0/37)
Oligodendrocyte-like cells	26% (11/43)	94% (17/18)	43% (16/37)
Neoplastic astrocytic component	98% (42/43)	11% (2/18)	59% (22/37)
Calcification	60% (26/43)	22% (4/18)	40% (15/37)
Necrosis	0% (0/43)	0% (0/18)	0% (0/37)
Microvascular proliferation	9% (4/43)	11% (2/18)	3% (1/37)
Rosenthal fibres	19% (8/43)	0% (0/18)	3% (1/37)
Eosinophilic granular bodies	49% (21/43)	0% (0/18)	3% (1/37)
Inflammation	51% (22/43)	0% (0/18)	19% (7/37)
Extravascular CD34 + (*n* = 76)	80% (28/35)	23% (3/13)	82% (23/28)
Cortical dysplasia (*n* = 59)	16% (4/25)	0% (0/9)	12% (3/25)

Clinical data were available for 69 GOSH patients for whom histological review had been carried out (32 GG, 14 DNET, 23 GNT NOS) (Table 2). 60 (87%) of these presented with seizures at diagnosis (25 GG, 13 DNET, 22 GNT NOS). This corresponded to 78% of ganglioglioma, 93% of DNET, and 96% of GNT NOS. These differences were not statistically significant by Fisher’s exact test (*p* = 0.125). Seizure follow-up data were available for all 60 of these patients. Mean follow-up duration was 25.3 months (range 1–96 months). Mean age at seizure onset was 55.8 months (range 3–188 months) for ganglioglioma, 78 months (range 24–168 months) for DNET, and 41.1 months (0.5–180 months) for GNT NOS. When assessed by ANOVA, there was no significant difference in age at seizure onset between the three groups (ANOVA *F* = 2.226, *p* = 0.117). Postoperatively, Fisher’s exact test showed that there was no significant difference in seizure freedom at 1 year or last follow-up across the three histological categories. 16/25 (64%) ganglioglioma were seizure free at 1 year, compared to 7/13 (54%) of DNET and 12/22 (54%) GNT NOS. At last follow-up, 15/25 (60%) ganglioglioma were seizure free, compared to 6/13 DNET (46%) and 12/22 (54%) GNT NOS. In total, 35/60 (58%) of patients were seizure free at 1 year postoperatively. This fell to 33/60 (55%) at last follow-up.

**Table 2:** Summary of clinical features for the 69 patients with glioneuronal tumours for which clinical data was available

Clinical features	Ganglioglioma	DNET	GNT NOS
Gender (*n* = 69)	19 M/13F	6 M/8F	11 M/12F
Presented with seizures (*n* = 69)	25 (*n* = 32)	13 (*n* = 14)	22 (*n* = 23)
Age 1st seizure (*n* = 60) (months)	56 (3–188) (*n* = 26)	78 (24–168) (*n* = 13)	24 (0.5–180) (*n* = 21)
Age surgery (*n* = 62) (months)	118 (20–201) (*n* = 26)	130 (41–188) (*n* = 13)	101 (17–212) (*n* = 23)
Seizure duration (*n* = 59) (months)	68 (8–162) (*n* = 25)	52 (1–146) (*n* = 13)	61 (5–165) (*n* = 21)
Residual post-op tumour (*n* = 55)	9 (*n* = 23)	7 (*n* = 12)	12 (*n* = 20)
Seizure free at 1 year (*n* = 60)	16 (*n* = 25)	7 (*n* = 13)	12 (*n* = 22)
Seizure free at last follow-up (*n* = 60)	15 (*n* = 25)	6 (*n* = 13)	12 (*n* = 22)

The following features were also analysed against seizure freedom at 1 year postoperatively and last follow-up: seizure duration prior to surgery, age at first seizure, age at surgery, and the presence of residual tumour on postoperative MRI. For all factors excluding residual tumour, we found no significant association at either 1 year or last follow-up. We identified a significant association between residual tumour and seizure outcome at 1 year (*p* = 0.046, Fisher’s exact test). Residual tumour was associated with a poorer rate of seizure freedom, although this effect was small and a large proportion of patients without residual tumour continued to experience seizures. 13/27 (48%) of patients with residual tumour were seizure free compared to 17/27 (63%) of patients without residual tumour. However, when assayed against seizure outcome at last follow-up, this association did not retain significance.

### RNA expression and DNA methylation identify two major groups of glioneuronal tumour

To investigate the presence of distinct biological entities within the spectrum of glioneuronal tumours, we utilised consensus clustering to undertake a class discovery approach to our RNA sequencing and methylation data.

**Fig. 1:**
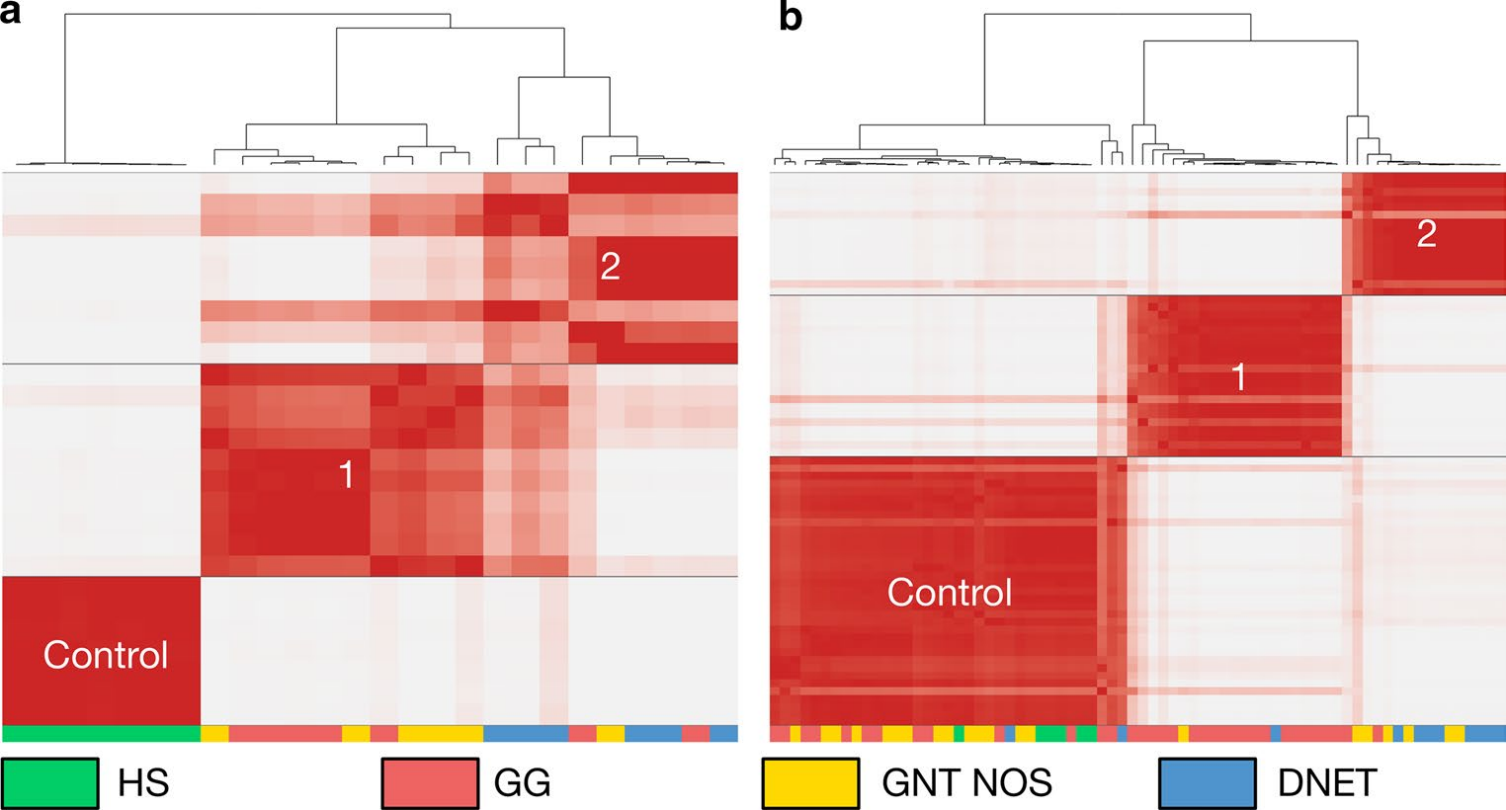
Glioneuronal tumours cluster into two groups, which are only partially consistent with histology, according to their expression and methylation profiles. **a** Consensus clustering heatmap of 19 tumours (7 GG 6 DNET, 6 GNT NOS) and 7 controls. Cases are clustered according to the top 5000 most variably expressed genes. Group 1 consisted of 5 GG and 5 GNT NOS (*n* = 10). Group 2 consisted of 2 GG, 6 DNET, and 1 GNT NOS (*n* = 9). **b** Clustering heatmap of 65 tumours (34 GG, 11 DNET, 20 GNT NOS) and 6 controls. Cases are clustered according to the top 10,000 most variably methylated CpGs. Group 1 consisted of 19 GG, 1 DNET and 1 GNT NOS (*n* = 21). Group 2 consisted of 2 GG, 8 DNET, and 6 GNT NOS (*n* = 16)

For the RNA sequencing cohort, after quality control to remove cases with low tumour content and poor-quality RNA, 19 tumours (7 GG, 6 DNET, 6 GNT NOS) and 7 controls were analysed. After inspecting the cumulative distribution function plot, we found that the clustering configuration that explained the most biological variability within the cohort represented three groups. The first of these consisted entirely of control cases (*n* = 7). The next, referred to as RNA Group 1, consisted of 5 ganglioglioma and 5 GNT NOS (*n* = 10). The final group, referred to as RNA Group 2, consisted of 6 DNET, 2 ganglioglioma, and 1 GNT NOS (*n* = 9). Clustering data for these cases are shown in Fig. 1a.

For the methylation profiling cohort, 65 tumours (34 GG, 11 DNET, 20 GNT NOS) and 6 controls were analysed. Recapitulating our observations from the RNA sequencing cohort, upon inspection of the cumulative distribution plot, we found that the clustering configuration that explained the most biological variability within the cohort represented three groups. The first of these consisted of all controls plus 28 tumours (13 GNT NOS, 13 GG, 2 DNET). The next, referred to as Methyl Group 1, consisted of 19 ganglioglioma, 1 DNET, and 1 GNT NOS (*n* = 21). The final group, referred to as Methyl Group 2, consisted of 8 DNET, 2 ganglioglioma, and 6 GNT NOS (*n* = 16). Clustering data for these cases are shown in Fig. 1b.

Our RNA and methylation cohorts were largely independent. 12 cases were subjected to both assays; however, only six of these had successfully clustered away from controls in both. These were six tumours that corresponded to RNA Group 2 in the expression data clustering, which also clustered with Methyl Group 2 during the methylation data clustering.

### Molecular subtype associates with age at seizure onset

**Fig. 2:**
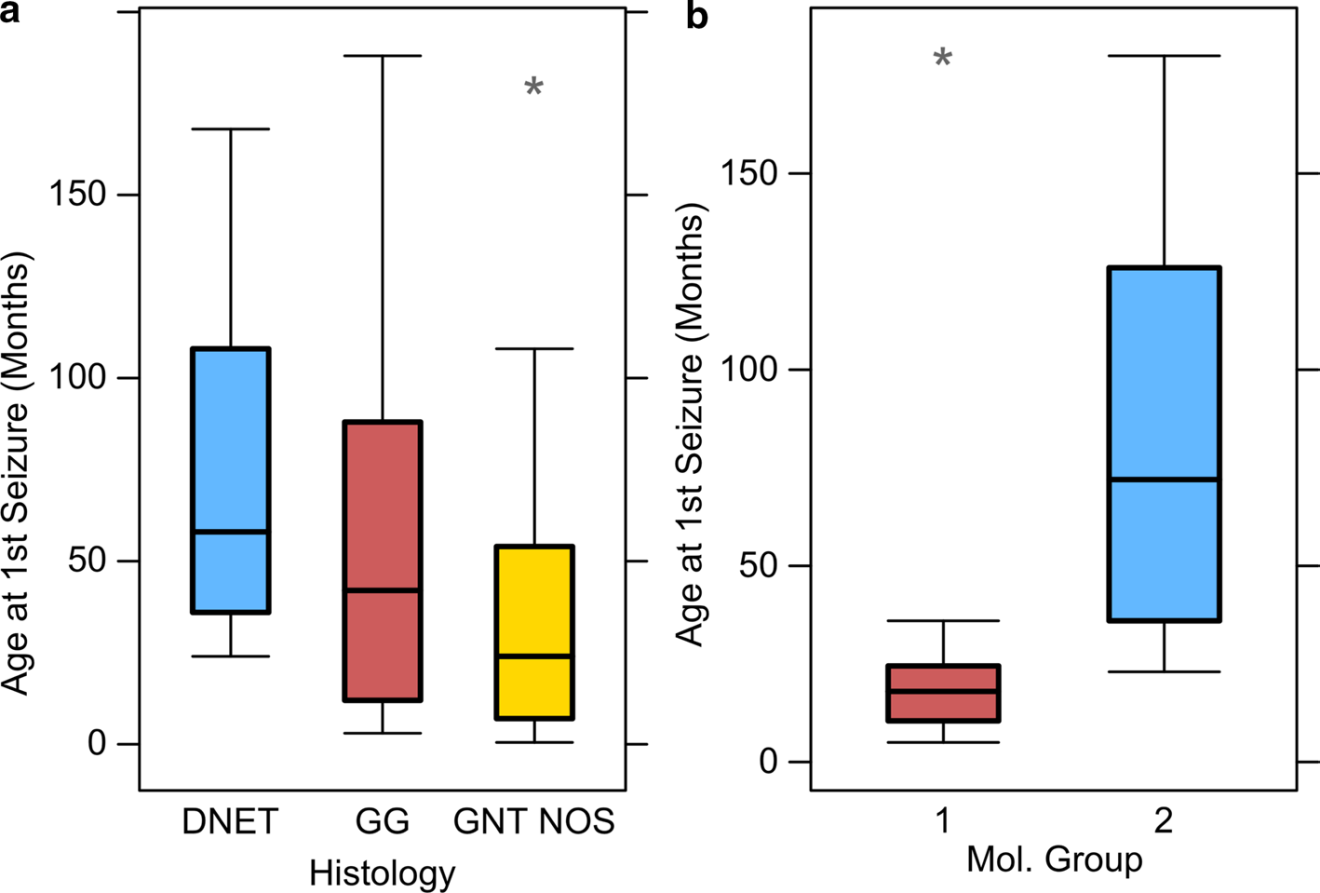
Molecular classification associates with age at first seizure. **a** When assayed against current histological classification, there is no significant association with age at first seizure (ANOVA *F* = 2.226, *p* = 0.117). **b** When tumours are classified according to their molecular profiles Group 1 tumours present with seizures significant earlier than Group 2 tumours (*t* = − 2.6, *p* = 0.017)

Having identified two distinct groups, we decided to analyse clinical features against molecular classification in the same manner as for histological classification. Seizure follow-up data were available for 28 cases that had been molecularly classified either by RNA or by methylation profiling (14 Group 1, 14 Group 2) (Table 3). Mean follow-up duration was 19.9 months (range 1.5–63 months). 12/14 (86%) Group 1 tumours presented with seizures, compared to 11/14 (79%) Group 2 tumours. We found no significant association between molecular group and seizure freedom at either 1 year postoperatively or last follow-up. 7/12 (58%) patients with Group 1 tumours and 5/11 (45%) patients with Group 2 tumours were seizure free at both 1 year and last follow-up. We also found no significant difference in seizure duration prior to surgery or age at surgery. However, we observed a striking difference in age at onset of seizures between the two groups (Fig. 2). Patients with Group 1 tumours possessed a mean age at first seizure of 30.2 months (SE = 13.9) compared to 87.5 (SE = 17.3) for those with Group 2 tumours (*t* = − 2.6, *p* = 0.017).

**Table 3:** Summary of clinical features for 28 tumours classified by expression or methylation profile

Clinical features	Group 1	Group 2
Gender (*n* = 28)	8 M/6F	7 M/7F
Presented with seizures (*n* = 28)	12 (*n* = 14)	11 (*n* = 14)
Age 1st seizure (months) (*n* = 28)	30 (5–180) (*n* = 14)	87 (23–180) (*n* = 14)
Age surgery (months) (*n* = 28)	99 (20–192) (*n* = 14)	141 (46–188) (*n* = 14)
Seizure duration (months) (*n* = 28)	67 (5–158) (*n* = 14)	55 (1–165) (*n* = 14)
Residual post-op tumour (*n* = 20)	7 (*n* = 9)	6 (*n* = 11)
Seizure free at 1 year (*n* = 23)	7 (*n* = 12)	5 (*n* = 11)
Seizure free at last follow-up (*n* = 23)	7 (*n* = 12)	5 (*n* = 11)

For the age data, the values are the mean and range

### Pathogenic mutations can be found in most tumour cases and segregate according to molecular subtype

To determine the genetic background of the two groups within the methylation and RNA sequencing cohorts, we decided to investigate the presence of pathogenic mutations by two methods.

**Fig. 3:**
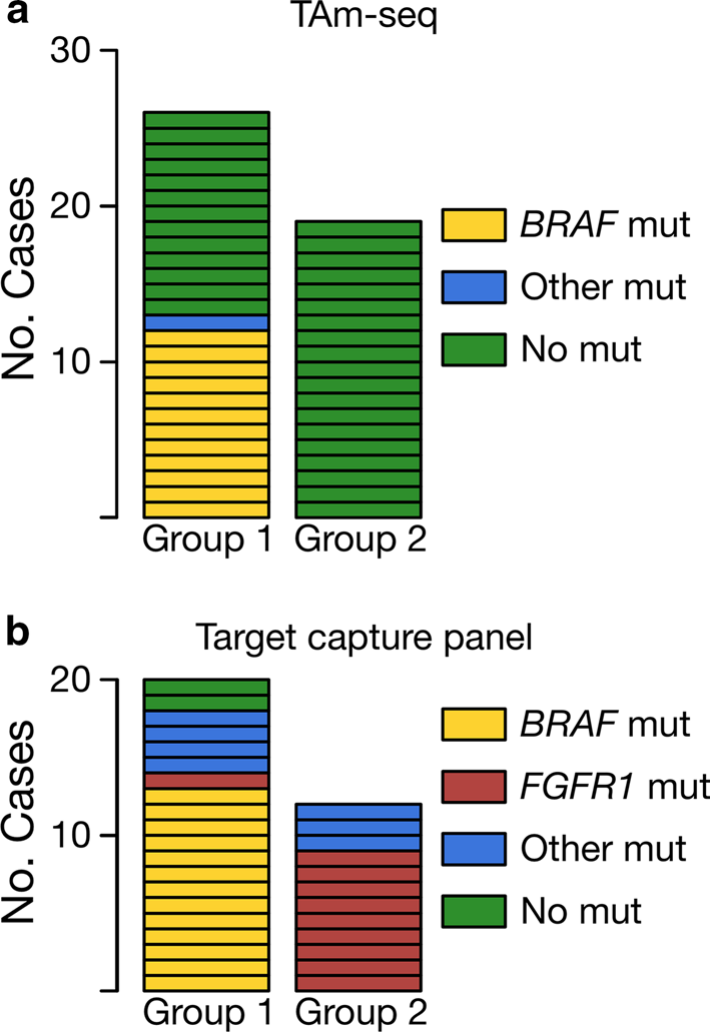
Majority of glioneuronal tumours possess pathogenic mutations; *BRAF* and *FGFR1* mutations are highly selective for groups identified by consensus clustering. **a** Tagged amplicon sequencing against 45 tumours assayed for mutations in *CTNNB1, BRAF, HIST1H3B, H3F3A, IDH1,* and *IDH2.* 14/45 (31%) possessed a mutation, of which the most common was *BRAF*-V600E in 12/26 (46%) Group 1 tumours. No *BRAF* mutations were detected in Group 2 tumours. Mutations in *CTNNB1, H3F3A, and IDH1* were detected in 4 cases, 1 of which was *BRAF*-V600E positive. **b** Target capture DNA sequencing against 32 tumours assayed for a panel of 78 genes. 30/32 (94%) possessed mutations. The most common abnormalities were *BRAF*-V600E, affecting 13/20 (65%) Group 1 tumours, and abnormalities in *FGFR1* in 1/20 (5%) Group 1 and 9/12 (75%) Group 2 tumours. Mutations in *ATM, TP53, AKT1, MAP2K2, HRAS, APC, WT1, ARID1A, ACVR1, MYCN,* and *CDK4* were also detected both independently and alongside *BRAF* and *FGFR1* abnormalities but were not recurrent

45 individual samples that had segregated from controls in the RNA sequencing and methylation cohorts underwent tagged amplicon sequencing (TAm-seq) and were assayed against a panel of six genes (Fig. 3a). These represented 26 RNA/Methyl Group 1 and 19 RNA/Methyl Group 2 tumours. Samples for which mutations were identified are detailed in Table 4. We observed abnormalities in 13/26 (54%) Group 1 tumours, of which 12 (46%) possessed a *BRAF*-V600E mutation. These were four cases from our RNA sequencing cohort and eight from our methylation cohort. One case with a *BRAF*-V600E mutation also possessed a *CTNNB1*-G34E mutation. The remaining mutation positive case possessed an *H3F3A*-K27M mutation. On histology review, this case was a ganglioglioma and the mutation was confirmed by immunohistochemistry. Of the 19 RNA/Methyl Group 2 tumours assayed, we identified no mutation positive cases. All variants detected are predicted to be pathogenic and have been previously described.

**Table 4:** Summary of mutation positive cases identified by tagged amplicon sequencing

Sample	Histology	Mol. group	Mutation	Depth	Mutation allele frequency		Average	Additional mutations
					Duplicate 1	Duplicate 2		
GNT03	GG	1	*BRAF*-V600E	207	8%	N/A	8%	N/A
GNT21	GG	1	*BRAF*-V600E	226	7%	8%	8%	N/A
GNT29	GG	1	*BRAF*-V600E	163	8%	N/A	8%	N/A
GNT35	GG	1	*BRAF*-V600E	1455	28%	32%	30%	N/A
GNT40	GNT NOS	1	*BRAF*-V600E	726	23%	25%	24%	N/A
GNT48	GG	1	*BRAF*-V600E	71	1%	1%	1%	N/A
GNT51	GG	1	*BRAF*-V600E	2948	23%	25%	24%	N/A
GNT52	GNT NOS	1	*BRAF*-V600E	79	7%	6%	7%	*CTNNB1*-G34E
GNT16	GG	1	*BRAF*-V600E	6370	17%	20%	19%	N/A
GNT01	GG	1	*BRAF*-V600E	6277	26%	23%	25%	N/A
GNT05	GG	1	*BRAF*-V600E	4400	18%	20%	19%	N/A
GNT17	GG	1	*BRAF*-V600E	2197	17%	18%	18%	N/A
GNT18	GG	1	*H3F3A*-K27M	3047	36%	24%	30%	N/A
GNT94	GG	N/A	*BRAF*-V600E	707	11%	13%	12%	N/A
GNT60	GG	N/A	*BRAF*-V600E	282	9%	8%	9%	N/A
GNT69	GG	N/A	*BRAF*-V600E	852	14%	15%	15%	N/A
GNT76	GG	N/A	*BRAF*-V600E	343	18%	11%	15%	N/A
GNT27	GG	N/A	*BRAF*-V600E	1403	19%	20%	20%	N/A
GNT95	GNT NOS	N/A	*BRAF*-V600E	1149	13%	15%	14%	N/A
GNT81	GNT NOS	N/A	*BRAF*-V600E	1482	14%	15%	15%	N/A
GNT39	GNTNOS	N/A	*BRAF*-V600E	5016	33%	N/A	33%	N/A
GNT96	GG	N/A	*BRAF*-V600E	2519	25%	25%	25%	N/A
GNT87	GNT NOS	N/A	*BRAF*-V600E	1097	11%	12%	12%	N/A
GNT89	GG	N/A	*BRAF*-V600E	1299	16%	16%	16%	N/A
GNT50	GNT NOS	N/A	*BRAF*-V600E	3185	30%	28%	29%	N/A
GNT54	GG	N/A	*BRAF*-V600E	558	17%	N/A	17%	N/A
GNT92	GNT NOS	N/A	*BRAF*-V600E	1694	18%	19%	19%	N/A
GNT44	GNT NOS	N/A	*BRAF*-V600E	196	34%	8%	21%	N/A

We also assayed the 28 tumours that had failed to segregate from controls in our methylation cohort by TAm-seq. We observed *BRAF*-V600E mutations in 15 (54%) of these cases. These mutation positive cases represented eight gangliogliomas and seven GNT NOS. We identified no other mutations in these tumours.

Having identified a number of mutation positive cases by TAm-seq, we decided to expand the scope of mutation screening using target capture sequencing against an existing panel of 78 genes developed at the Institute of Cancer Research. 32 individual samples from our RNA sequencing and methylation cohorts underwent mutation screening (Fig. 3b). These represented 20 RNA/Methyl Group 1 and 12 RNA/Methyl Group 2 tumours. Samples for which mutations were identified are detailed in Table 5. We observed abnormalities in 18/20 (90%) of Group 1 tumours, of which 13 (65%) possessed a *BRAF*-V600E mutation. These were five cases from our RNA sequencing cohort and eight from our methylation cohort. Six cases with *BRAF*-V600E mutations possessed additional mutations. The remaining five mutation positive Group 1 tumours lacking a *BRAF* mutation possessed an *FGFR1, NF1, ARID1B, HIST1H3B,* and *ATM* mutations, respectively.

**Table 5:** Summary of mutation positive cases identified by target capture DNA sequencing

Sample	Histology	Mol. group	Depth	*BRAF/FGFR1*	Additional mutations
GNT03	GG	1	682	*BRAF*-V600E (12%)	*ARID1B*-Q717K (49%)
GNT15	GG	1	432	*BRAF*-V600E (12%)	
GNT22	GG	1	37	*BRAF*-V600E (15%)	*CTNNB1*-A39V (10%)
GNT16	GG	1	698	*BRAF*-V600E (20%)	*ASXL1*-R235W (42%)
GNT21	GG	1	264	*BRAF*-V600E (22%)	
GNT51	GG	1	677	*BRAF*-V600E (24%)	*ASXL1*-S1428P (51%)
GNT40	GNT NOS	1	1051	*BRAF*-V600E (25%)	
GNT17	GG	1	518	*BRAF*-V600E (26%)	
GNT35	GG	1	557	*BRAF*-V600E (32%)	*FGFR2*-Q779A (46%), *CDNK2A/B* Del, *ATM*-R2461C (20%)
GNT29	GG	1	20	*BRAF*-V600E (50%)	
GNT45	GG	1	14	*BRAF*-V600E (50%)	
GNT18	GG	1	708	*BRAF*-V600E (1%)	*H3F3A*-K27M (29%)
GNT09	GG	1	185	*BRAF*-V600E (4%)	
GNT37	DNET	2	953	*FGFR1*-A334T (32%)	
GNT31	DNET	2	258	*FGFR1*-L567E (36%)	*ATM*-R1039L (9%)
GNT07	GNT NOS	2	711	*FGFR1* duplication	
GNT23	DNET	1	414	*FGFR1* duplication	*MLL2*-R3596W (43%)
GNT33	DNET	2	410	*FGFR1* duplication	
GNT10	DNET	2	714	*FGFR1* duplication	
GNT43	DNET	2	1153	*FGFR1* duplication	*TP53*-R282Q (47%), N235S (44%)
GNT38	DNET	2	1021	*FGFR1* duplication	*AKT1*-E117 Del (37%)
GNT14	GNT NOS	2	173	*FGFR1* duplication	
GNT24	DNET	2	488	*FGFR1* e18 Inversion	*MAP2K2*-S127L (46%)
GNT36	GG	2	1568	N/A	*HRAS*-A134V (48%), *APC*-E1209K (44%), *WT1*-G60R (38%)
GNT13	GG	1	974	N/A	*NF1*-L585 Frameshift (54%)
GNT49	GG	2	791	N/A	*ARID1A*-158S (72%), *ACVR1*-V435 Del (42%)
GNT11	GG	1	7	N/A	
GNT08	GG	1	1251	N/A	*ARID1B*-H92L (38%)
GNT42	GG	1	607	N/A	*HIST1H3B*-M121T (47%)
GNT30	GG	2	39	N/A	*MYCN* + *CDK4* Amplified
GNT47	GNT NOS	1	712	N/A	*ATM*-V2696L (48%)

We identified mutations in all 12 Group 2 tumours that were assayed by target capture DNA sequencing. Of these, 9/12 (75%) possessed an *FGFR1* mutation. These *FGFR1* mutations consisted of 6 tandem duplications, 1 exon 18 inversion, and 1 instance each of A334T and L567E substitutions. The latter two of these are not previously described to our knowledge. These cases represented 5 that had been in both our RNA and methylation cohorts and 4 from our methylation cohort alone. 4 Group 2 tumours that were positive for an *FGFR1* mutation also possessed additional abnormalities. These were mutations in *ATM*, *TP53, AKT1,* and *MAP2K2*. The remaining three cases lacking an *FGFR1* abnormality were 1 case possessing *HRAS, APC,* and *WT1* mutations, 1 case possessing *ARID1A* and *ACVR1* mutations, and 1 case with *MYCN* and *CDK4* amplifications. While all cases were screened against commercial genomic DNA, and ExAC and dbSNP databases, it should be noted that all variants identified by this assay that are non-recurrent across the cohort may represent private SNPs rather than pathogenic mutations.

### Copy number changes are frequent, but are rarely recurrent

Copy number status can be estimated from methylation data, and copy number abnormalities have previously been reported in glioneuronal tumours. Therefore, we decided to investigate the presence of copy number abnormalities across the two molecular groups we had identified. In total, we assayed 65 tumours and found copy number abnormalities in 17 (26%) (Table 6). Histologically, these represented nine ganglioglioma, three DNET, and five GNT NOS. When labelled according to molecular classification, these were 7 Group 1 tumours and 4 Group 2 tumours. In addition, we identified copy number abnormalities in six tumours that had not classified robustly with either group away from controls. We identified copy number abnormalities in all chromosomes except 3 and 16. Losses were present in chromosomes 1, 2, 4, 8, 9, 10, 11, 12, 13, 14, 17, 19, and 22. Gains were present in chromosomes 1, 5, 6, 7, 8, 10, 11, 12, 15, 18, 19, 20, and 21. The most common abnormalities across the cohort were gain of chromosome 7 (*n* = 7) and loss of chromosome 13 (*n* = 6). Chromosome 22 loss was also observed in 4 cases. No copy number change was significantly associated with molecular or histological classification. In addition, neither *BRAF* nor *FGFR1* mutation status associated with copy number changes.

**Table 6:** Summary of copy number abnormalities found in 17/65 glioneuronal tumours

Sample	Histology	Mol. group	Losses	Gains
GNT42	GG	1	8, 13	
GNT57	GG	1		7
GNT58	GG	1		5, 7
GNT16	GG	1	2, 13, 19, 22	20
GNT17	GG	1	1	
GNT35	GG	1	9, 10, 11, 13, 14, 22	
GNT47	GNT NOS	1		1,7
GNT59	DNET	N/A	4, 10, 12, 13, 17	7, 19
GNT04	GG	N/A		7, 12, 19, 20
GNT27	GG	N/A	13	
GNT39	GNT NOS	N/A		5, 7, 8, 11, 12, 15, 18, 20
GNT46	GG	N/A	10	
GNT50	GNT NOS	N/A	9	
GNT02	GNT NOS	2	9, 13	
GNT07	DNET	2		21
GNT25	GNT NOS	2	22	
GNT37	DNET	2	22	5, 6, 7, 10, 13

In Mol. Group, N/A are cases that clustered with controls during consensus clustering

### The molecular subtypes show different patterns of cytological differentiation

To test the hypothesis that the two molecular groups were composed of distinct cell types, we used gene set enrichment analysis to assay our RNA sequencing cohort for differential enrichment of genes, whose expression is associated with specific neural cell types (Table 7). When compared against controls, we observed enrichments in Group 1 tumours corresponding to astrocytic, microglial, and endothelial phenotypes (*p* > 0.05). When we compared Group 2 tumours to controls, we identified enrichments for endothelial, microglial, and oligodendrocyte precursor phenotypes (*p* > 0.05). When we compared Group 1 tumours vs Group 2 tumours directly, we identified enrichments for astrocytic and neuronal phenotypes in Group 1 tumours. Group 2 tumours were enriched for endothelial and all oligodendroglial lineage phenotypes, with the oligodendrocyte precursor phenotype demonstrating the strongest score.

**Table 7:** Gene set enrichment analysis for neural-cell-type specific gene sets

Gene Set	Size	ES	NES	*p* value	*q* value
Group 1 vs Control					
ASTROCYTE	149	0.68	2.98	< 0.0005	< 0.0005
MICROGLIA	148	0.62	2.72	< 0.0005	< 0.0005
ENDOTHELIA	148	0.59	2.57	< 0.0005	< 0.0005
MO	149	− 0.73	− 3.31	< 0.0005	< 0.0005
NFO	148	− 0.69	− 3.13	< 0.0005	< 0.0005
NEURON	148	− 0.53	− 2.40	< 0.0005	< 0.0005
OPC	149	− 0.32	− 1.47	0.005	0.005
Group 2 vs Control					
ENDOTHELIA	149	0.71	3.06	< 0.0005	< 0.0005
MICROGLIA	148	0.61	2.64	< 0.0005	< 0.0005
OPC	149	0.61	2.64	< 0.0005	< 0.0005
ASTROCYTE	149	0.39	1.68	< 0.0005	0.001
MO	149	− 0.63	− 2.71	< 0.0005	< 0.0005
NEURON	148	− 0.61	− 2.53	< 0.0005	< 0.0005
NFO	148	− 0.46	− 1.96	< 0.0005	< 0.0005
Group 1 vs Group 2					
ASTROCYTE	149	0.61	2.74	< 0.0005	< 0.0005
NEURON	148	0.40	1.76	< 0.0005	< 0.0005
MO	149	− 0.37	− 1.64	< 0.0005	< 0.0005
NFO	148	− 0.52	− 2.28	< 0.0005	< 0.0005
ENDOTHELIA	149	− 0.52	− 2.32	< 0.0005	< 0.0005
OPC	149	− 0.72	− 3.22	< 0.0005	< 0.0005

*ES* enrichment score. *NES* normalised enrichment score. For these scores, the highest and lowest scores are the most strongly enriched within each of the groups being compared, respectively. *MO* mature oligodendrocyte.* NFO* newly formed oligodendrocyte. *OPC* oligodendrocyte precursor cell

We were able to perform a similar gene set enrichment analysis on the two molecular groups within our methylation cohort, with the aim of confirming differential enrichment of astrocyte and oligodendrocyte precursor phenotypes between groups. When we compared Group 1 vs Group 2 tumours, we observed differential enrichments for both oligodendrocyte precursor and astrocyte gene sets within the list of differentially methylated sites between groups (*p* > 0.005) (Online Resource 3).

**Fig. 4:**
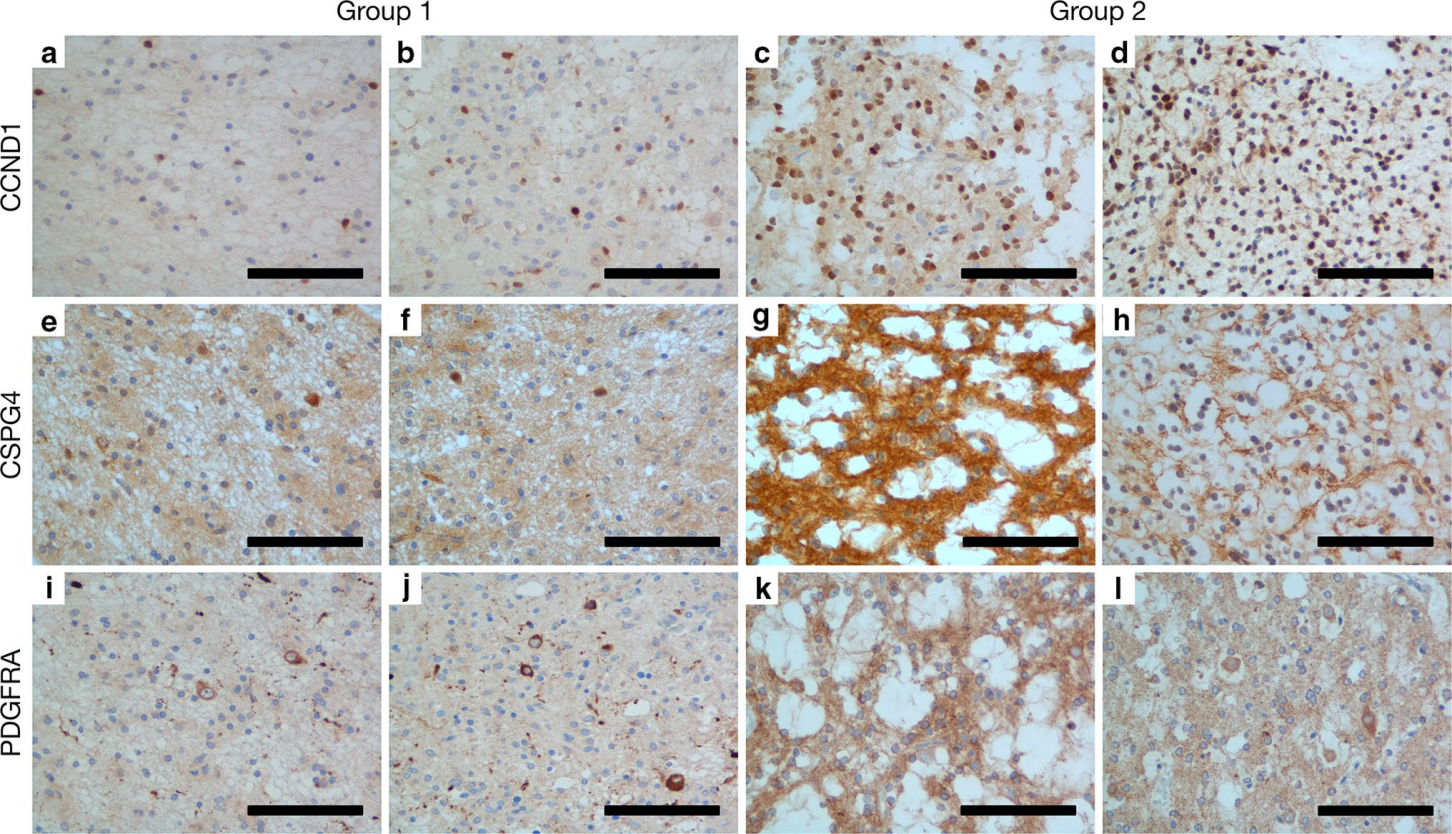
Immunohistochemistry against CCND1 (**a**–**d**), CSPG4 (**e**–**h**), and PDGFRA **(i-l)** in Group 1 and Group 2 tumours. CCND1 displayed the most striking difference between tumour groups, staining a minority of tumour cells within Group 1 tumours, in contrast to a majority in Group 2 tumours. CSPG4 was more variable between cases within groups and therefore less robust, staining neurons in Group 1 tumours, compared to a proportion of “oligodendrocyte-like” cells and the neuropil in Group 2 tumours. For PDGFRA we observed a thin rim of membranous/cytoplasmic staining in the majority of tumour cells within Group 2 tumours, while only neurons were stained in Group 1 tumours. Scale bars = 100 μm

**Fig. 5:**
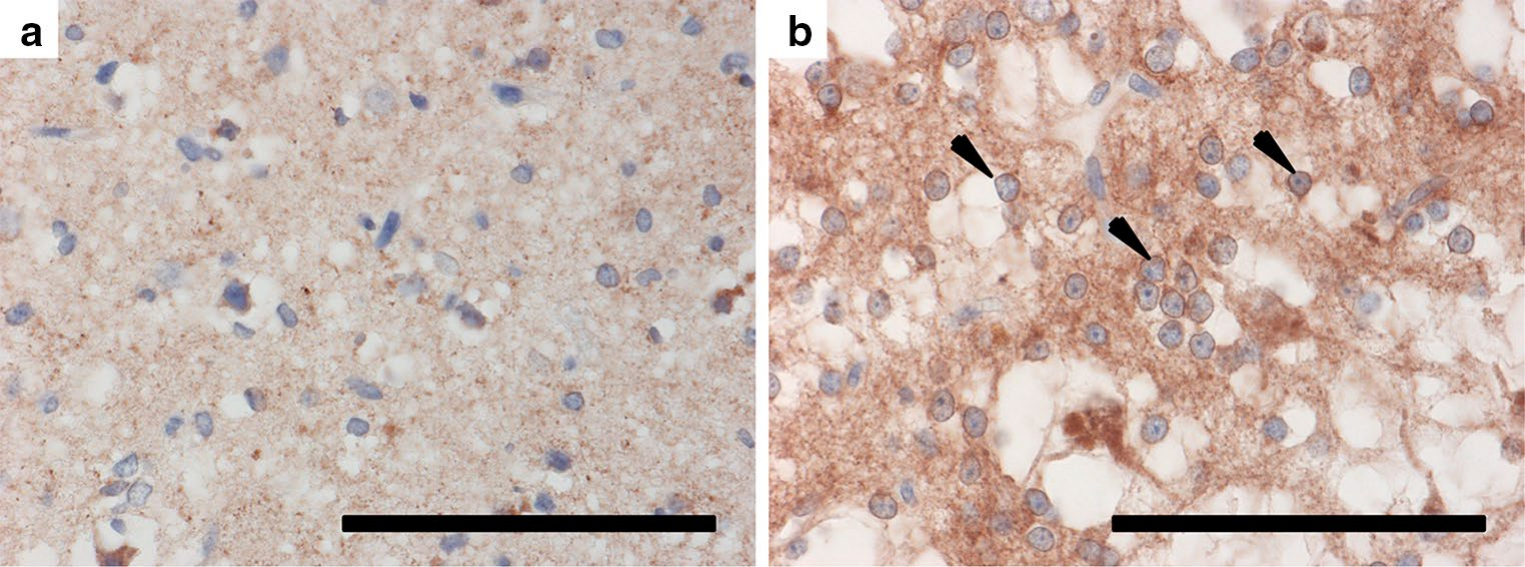
Immunohistochemistry against PDGFRA in a Group 1 and Group 2 tumour. **a** PDGFRA staining is largely absent in Group 1 tumours. **b** Thin rim of staining can be observed in the tumour cells of Group 2 tumours (arrows). Scale bars = 100 μm

Having observed an oligodendrocyte precursor enrichment in Group 2 tumours as part our gene set enrichment analysis, we hypothesised that immunohistochemistry directed against oligodendrocyte precursors could distinguish the tumour groups. We selected CCND1, CSPG4, and PDGFRA as targets due to their association with this cell type (Fig. 4). Five cases from each group were randomly selected and stained for all three targets (total *n* = 10). For CCND1, we noted a striking difference in staining between groups. In Group 1 tumours, only a small proportion of tumour cells were weakly stained, compared to a majority of tumour cells possessing moderate-to-strong staining in Group 2 tumours (Fig. 4a–d). This pattern was repeated for PDGFRA, for which we observed a thin rim of cytoplasmic/membranous staining in the majority of tumour cells within Group 2 tumours, while only neurons were stained in Group 1 tumours (Figs. 4i–l,  5). For both CCND1 and PDGFRA, staining within Group 2 tumours was most prominent within the “oligodendrocyte-like” cell population often described within DNETs. CSPG4 displayed a less striking difference between tumour groups. Staining was slightly more prominent in Group 2 tumours, in particular with moderate-to-strong staining in the cell membrane of a proportion of “oligodendrocyte-like” cells and the neuropil. However, staining was highly variable between cases. In contrast, within Group 1 tumours, staining was evident only in neurons and a fraction of tumour cells (Fig. 4e–h).

To validate the utility of these targets in distinguishing tumours, cases were reviewed and segregated into two groups by an independent pathologist (WM), blinded to molecular classification and prior histological diagnosis (Online Resource 4). CCND1 was the best performing stain, for which all cases (10/10) were segregated concordantly with molecular classification. 9/10 cases stained with CSPG4 were segregated in agreement with molecular classification. The remaining case, a histologically difficult case with limited tumour content and extensive calcification, could not be confidently segregated. PDGFRA performed similarly, with 8/10 cases segregated in a manner concordant with molecular classification. The remaining two cases were two Group 2 tumours that were segregated alongside Group 1 tumours.

## Discussion

Glioneuronal tumours are poorly distinguished by their histological features and a proportion of cases possess uninformative histological appearances that preclude confident classification. Interestingly, 37% of the cases within our GOSH cohort did not fit well within the current histological subtypes. However, this problem is not unique to our practice or diagnostic criteria as poor inter-observer correlation is commonplace in published studies, particularly for ganglioglioma and DNET, for which there is unexplained and marked geographical variability across surgical series (Online Resource 1) [16]. In this context, improving the consensus for histopathological diagnosis forms part of an on-going debate, with a focus particularly directed towards increased use of molecular genetic assays [2]. Our data suggest that most glioneuronal tumours fall within two major groups as defined by expression profiling and DNA methylation. These groups are only partially consistent with the existing histological classification, and each includes a number of tumours for which traditional histological examination proved insufficient. This, therefore, suggests that the histological appearance of these tumours may not robustly reflect their underlying biology. Because of this, we favour the development of classification strategies based on biological rather than histological similarity.

While the majority of tumours could be segregated by molecular classification into two groups, a number could not be distinguished cleanly from controls. This was particularly true for our methylation analysis, in which 28/65 tumour samples clustered with control temporal cortex. While it is possible that these may represent a third group of tumours, it seems more likely that these simply represent those with low tumour content and consequently weaker methylation profiles, particularly given the architectural pattern of some glioneuronal tumours that intermix with normal cells. This is supported by the identification of *BRAF* mutations in a number of cases that failed to segregate, which likely represent Group 1 tumours when taken together with the observed enrichment of *BRAF* mutations within this group. An alternative explanation for the failure to segregate, given that all DNA was derived from FFPE material, may be that these samples represent those with significant DNA degradation, distorting their methylation profiles, and making them difficult to distinguish. As degradation is likely to be uneven across the genome, this may leave specific sites relatively intact, explaining why we were able to detect mutations in a large proportion of these tumours.

In each molecular group, we identified a high proportion of cases for which pathogenic mutations could be identified (Online Resource 5). Group 1, containing a relatively high proportion of tumours with a ganglioglioma-like appearance, is enriched for *BRAF*-V600E mutations. Group 2 tumours, with approximately half of the tumours displaying a DNET-like appearance, are enriched for *FGFR1* mutations. This is partially consistent with the previous reports of mutations within glioneuronal tumours. Qaddoumi et al. recently identified *BRAF* mutations in 53% of ganglioglioma, with V600E mutations accounting for 35% of cases, and *FGFR1* mutations in 82% of DNETs [13]. In addition, Rivera et al. were able to identify *FGFR1* mutations in 58% of DNETs [14]. However, *BRAF* mutations and copy number abnormalities have also been reported in DNETs, complicating the genetic background of these tumours and making consensus difficult [4, 7, 10]. A specific feature of these studies is that they segregated tumours according to histological classification, which our data suggest may not be reliable or reflective of the underlying biology. Having taken a novel and unbiased approach to classification, our findings suggest that *BRAF* and *FGFR1* mutations are highly specific within glioneuronal tumours to each of the two molecular tumour groups we have identified, and may be well suited as markers for distinguishing them from one another. The specificity of these mutations to each group also supports the robustness of our consensus clustering approach. It is curious that a proportion of cases seemingly lack these mutations. However, given the low average allele frequency of these mutations across the cohort, it is possible that they may be present at such low frequencies in some cases that they may not necessarily be detected without multiple assays to avoid under-sampling. In cases possessing mutations, the relatively low and variable mutation allele frequencies across the cohort are likely explained by the architecture of these tumours, a large proportion of which present as nodules of abnormal cells admixed with cells that appear histologically normal. Alternatively, it may be that only specific populations of tumour cells harbour the mutation. Supporting this hypothesis, mutant BRAF expression in ganglioglioma has previously been reported to associate with the neuronal component [8]. For cases where mutations cannot be detected, classification may be achievable through genomic profiling by methylation arrays, or by the use of immunohistochemical stains such as CCND1, which we found displayed the most robust and consistent difference in staining between the two molecular groups.

In addition to the identification of mutations in each tumour group, we were able to demonstrate enrichment for specific cell types. Group 1 was enriched most prominently for an astrocytic phenotype, while Group 2 was enriched for an oligodendrocyte precursor phenotype, indicating contrasting cellular differentiation between the two tumour groups. The latter of these was validated by our immunohistochemical assays against the oligodendrocyte precursor-associated proteins CCND1, CSPG4, and PDGFRA. This novel finding may potentially indicate differing cells of origin for each of the tumour types. However, it is also a possibility that these phenotypes represent a downstream effect of the mutations enriched within each group. *BRAF*-V600E activity has previously been associated with astrocyte hyper-proliferation, while *FGFR1* activity is associated with inhibition of oligodendrocyte precursor differentiation [17, 20]. If these mutations occur in a common precursor and drive tumour development by influencing differentiation direction, then this may explain why Group 1 tumours, enriched for *BRAF*-V600E mutations display an astrocytic expression phenotype, while Group 2 tumours display an oligodendrocyte precursor enrichment alongside frequent *FGFR1* mutations.

While we observed no difference in seizure outcome between groups for patients presenting with epilepsy, we noted a significant difference in the age at which seizures first arise. This may suggest a difference in the early clinical course of these tumours, and supports the hypothesis that the two groups identified by expression and methylation profiling are indeed distinct biological entities rather than categorisation by chance.

**Fig. 6:**
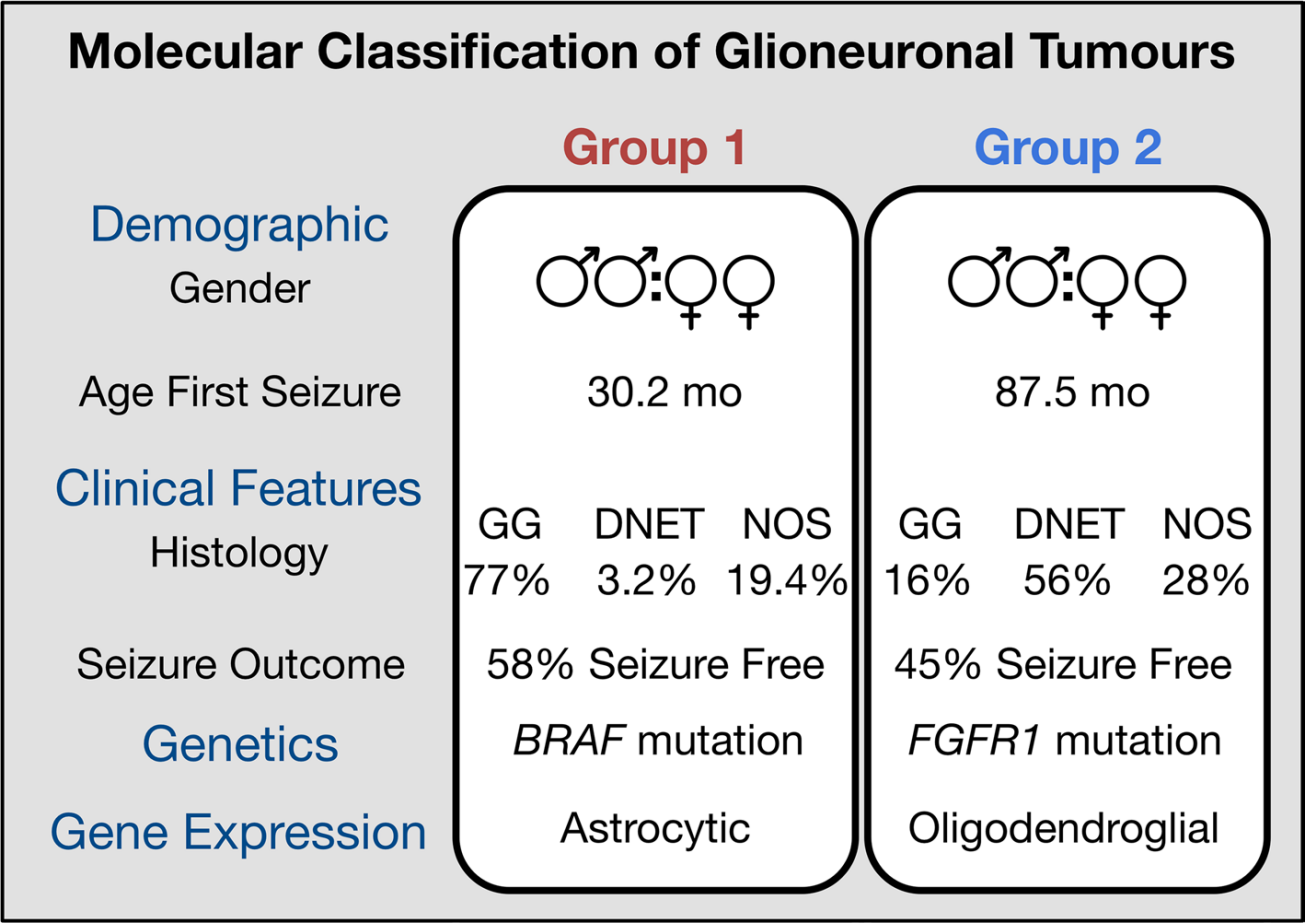
Molecular classification of glioneuronal tumours. Glioneuronal tumours can be classified into two groups based on their molecular profiles. These groups are only partially consistent with existing histological classification. Group 1 tumours are enriched for *BRAF* mutations and possess an astrocytic expression phenotype. In addition, they present with seizures significantly earlier. In contrast, Group 2 tumours are enriched for *FGFR1* mutations and possess an oligodendrocyte precursor expression phenotype

In summary, we present a new model for the classification of glioneuronal tumours, based on biological similarity rather than subjective histological examination (Fig. 6). We have shown that glioneuronal tumours can be classified into two groups by either their expression or methylation profiles. This is particularly useful for cases, where the histological features are uninformative and confident diagnosis is precluded (GNT NOS). Our classification is supported by the identification of *BRAF* and *FGFR1* mutations, which are strongly associated with each group, and the differential enrichment of distinct neural cell phenotypes: astrocytic for Group 1 and oligodendroglial for Group 2. This biologically guided classification should help towards addressing the marked diagnostic variability seen across surgical series and may assist in refining future cohorts for which accurate segregation of biological entities is necessary.

## Electronic supplementary material

Below is the link to the electronic supplementary material.

Supplementary material 1 (PDF 1015 kb)
