# Supplementary material for: Comprehensive molecular characterisation of epilepsy-associated glioneuronal tumours
Source: Acta Neuropathol. 2017 Oct 20;135(1):115–29. doi: 10.1007/s00401-017-1773-z (PMC5756244; doi:10.1007/s00401-017-1773-z)
Supplement: Supplementary file 1 — Supplementary material 1 (PDF 1015 kb) [file 401_2017_1773_MOESM1_ESM.pdf]

Comprehensive molecular characterisation of epilepsy-associated glioneuronal tumours

Acta Neuropathologica

Thomas J Stone[1,4], Angus Keeley[2], Alex Virasami[4], William Harkness[5], Martin Tisdall[5], Elisa Izquierido Delgado[9], Alice Gutteridge[8], Tony Brooks[3], Mark Kristiansen[3], Jane Chalker[7], Lisa Wilkhu[7], William Mifsud[4], John Apps[1], Maria Thom[11], Mike Hubank[10], Tim Forshew[8], J Helen Cross[2], Darren Hargrave[6], Jonathan Ham[1], Thomas S Jacques[1,4]

Developmental Biology and Cancer Programme[1], Developmental Neuroscience Programme[2] and UCL Genomics[3], UCL Great Ormond Street Institute of Child Health, London WC1N 1EH, UK

Departments of Histopathology[4], Neurosurgery[5], Haematology and Oncology[6] and Haematology, Cellular and Molecular Diagnostics Service[7], Great Ormond Street Hospital for Children NHS Foundation Trust, Great Ormond Street, London WC1N 3JH, UK

Department of Pathology[8], UCL Cancer Institute, London, WC1E 6DD

Glioma Team, Division of Molecular Pathology and Cancer Therapeutics[9], The Institute of Cancer Research, London, SM2 5NG

Centre for Molecular Pathology[10], Royal Marsden Hospital, London, SM2 5NG

Department of Clinical and Experimental Epilepsy[11], UCL Institute of Neurology, London, WC1N 3BG

Author for correspondence:
Dr Thomas S Jacques
Email: t.jacques@ucl.ac.uk

Contents:

Online Resource 1: Incidence of glioneuronal tumours across epilepsy surgical series. Reported frequencies for ganglioglioma, DNET, and mixed glioneuronal tumours (DNET/GG & GNT NOS) in large epilepsy surgical series demonstrate significant unexplained variability. Adapted from Thom *et al.* [15]

Online Resource 2: Cumulative distribution function delta area plots for consensus clustering of expression **(a)** and methylation **(b)** data. To determine the optimum number of groups within each cohort, the k corresponding to the first downwards inflection in the cumulative distribution function plot was used. This represents the point at which further subdividing the cohort into additional groups explains little additional variability in the biological data.

Online Resource 3: Gene set enrichment statistics for the comparison of astrocytic and oligodendrocyte precursor gene sets between Methyl Group 1 and 2 tumours. N = Number of genes within the gene set. DE = Number of differentially methylated gene set genes.

Online Resource 4: Segregation of cases by immunohistochemistry for CCND1, CSPG4, and PDGFRA and concordance with molecular classification. Cases were reviewed and segregated into two groups by a pathologist blinded to molecular classification and histological diagnosis.

Online Resource 5: Summary of molecular findings for all cases classified by RNA sequencing and 450k methylation array.

Online Resource 1: Incidence of glioneuronal tumours across epilepsy surgical series. Reported frequencies for ganglioglioma, DNET, and mixed glioneuronal tumours (DNET/GG & GNT NOS) in large epilepsy surgical series demonstrate significant unexplained variability. Adapted from Thom *et al.* [16]

**Series**	**Total No.**	**No. GNT**	**DNET**	**Ganglioglioma**	**DNET/GG**	**GNT NOS**
NHNN, London (Adult)	155	125	88 (57%)	12 (8%)	5 (3%)	10 (6%)
Kings, London	92	80	74 (80%)	6 (7%)	N/A	N/A
Grenoble	94	90	61 (65%)	29 (31%)	N/A	N/A
Cleveland (Adult)	141	65	10 (7%)	38 (27%)	N/A	14 (10%)
Cleveland (Paed.)	129	88	17 (13%)	48 (37%)	N/A	18 (14%)
Beijing	51	42	10 (20%)	19 (37%)	N/A	13 (25%)
Illinois	39	24	10 (26%)	14 (36%)	N/A	N/A
GEBB, Erlangen	1354	972	246 (18%)	669 (49%)	5 (0.4%)	52 (4%)

Online Resource 2: Cumulative distribution function delta area plots for consensus clustering of expression **(a)** and methylation **(b)** data. To determine the optimum number of groups within each cohort, the k corresponding to the first downwards inflection in the cumulative distribution function plot was used. This represents the point at which further subdividing the cohort into additional groups explains little additional variability in the biological data.

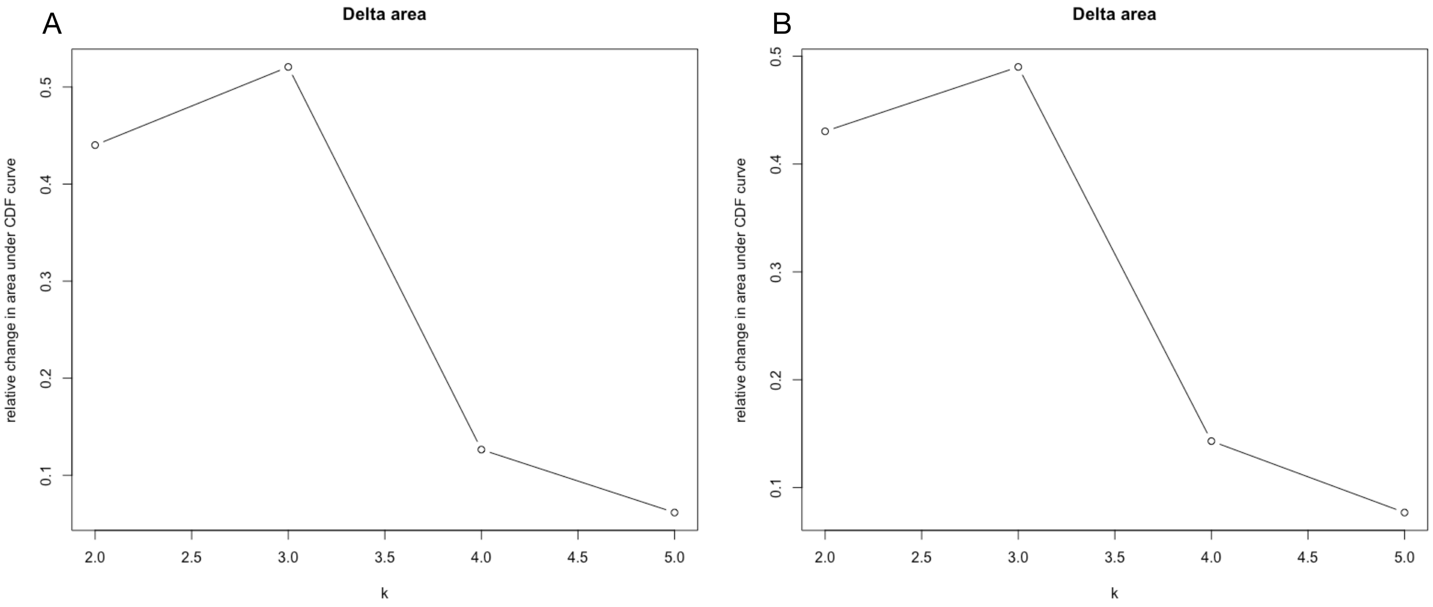

Online Resource 3: Gene set enrichment statistics for the comparison of astrocytic and oligodendrocyte precursor gene sets between Methyl Group 1 and 2 tumours. N = Number of genes within the gene set. DE = Number of differentially methylated gene set genes.

Gene set	N	DE	p-value	q-value
Astrocyte	150	140	> 0.005	> 0.005
OPC	150	139	> 0.005	> 0.005

Online Resource 4: Segregation of cases by immunohistochemistry for CCND1, CSPG4, and PDGFRA and concordance with molecular classification. Cases were reviewed and segregated into two groups by a pathologist blinded to molecular classification and histological diagnosis.

	GNT51	GNT15	GNT40	GNT52	GNT21	GNT38	GNT10	GNT43	GNT37	GNT28
Mol. Class	1	1	1	1	1	2	2	2	2	2
CCND1	1	1	1	1	1	2	2	2	2	2
CSPG4	1	1	1	1	1	2	2	2	2	N/A
PDGFRA	1	1	1	1	1	2	2	2	1	1

Online Resource 5: Summary of molecular findings for all cases classified by RNA sequencing and 450k methylation array.

Sample	Histology	RNA/Meth	Mol. Group	Mutations
GNT21	GG	RNA	RNA 1	*BRAF*-V600E
GNT41	GG	RNA	RNA 1	N/A
GNT45	GG	RNA	RNA 1	*BRAF*-V600E
GNT51	GG	RNA	RNA 1	*BRAF*-V600E, *ASXL1*-S1428P
GNT15	GG	RNA	RNA 1	*BRAF*-V600E
GNT19	GNT NOS	RNA	RNA 1	*N/A*
GNT40	GNT NOS	RNA	RNA 1	*BRAF*-V600E
GNT44	GNT NOS	RNA	RNA 1	*N/A*
GNT47	GNT NOS	RNA	RNA 1	*ATM*-V2696L
GNT52	GNT NOS	RNA	RNA 1	*BRAF*-V600E, *CTNNB1*-G34E
GNT32	DNET	RNA	RNA 2	*N/A*
GNT30	GG	RNA	RNA 2	*MYCN* + *CDK4* Amplified
GNT28	GNT NOS	RNA	RNA 2	*N/A*
GNT43	DNET	RNA + Meth	RNA 2: Meth 2	*FGFR1* Duplication, *TP53*-R282Q, *TP53*-N235S
GNT10	DNET	RNA + Meth	RNA 2: Meth 2	*FGFR1* Duplication
GNT37	DNET	RNA + Meth	RNA 2: Meth 2	*FGFR1*-A334T
GNT38	DNET	RNA + Meth	RNA 2: Meth 2	*FGFR1* Duplication, *AKT1*-E117 Del
GNT24	DNET	RNA + Meth	RNA 2: Meth 2	*FGFR1* E18 Inversion, *MAP2K2*-S127L
GNT36	GG	RNA + Meth	RNA 2: Meth 2	*HRAS-A134V, APC-E1209K, WT1-G60R*
GNT01	GG	Meth	Meth 1	*BRAF*-V600E
GNT03	GG	Meth	Meth 1	*BRAF*-V600E, *ARID1B*-Q17K
GNT05	GG	Meth	Meth 1	*BRAF*-V600E
GNT06	GG	Meth	Meth 1	*N/A*
GNT08	GG	Meth	Meth 1	*ARID1B*-H92L
GNT09	GG	Meth	Meth 1	*BRAF*-V600E
GNT11	GNT NOS	Meth	Meth 1	*N/A*
GNT12	GG	Meth	Meth 1	*N/A*
GNT13	GG	Meth	Meth 1	*NF1*-L585 Frameshift
GNT16	GG	Meth	Meth 1	*BRAF*-V600E, *ASXL1*-R235W
GNT17	GG	Meth	Meth 1	*BRAF*-V600E
GNT18	GG	Meth	Meth 1	*BRAF*-V600E, *H3F3A*-K27M
GNT22	GG	Meth	Meth 1	*BRAF*-V600E, *CTNNB1*-A39V
GNT23	DNET	Meth	Meth 1	*FGFR1* Duplication, *MLL2*-R3596W
GNT29	GG	Meth	Meth 1	*BRAF*-V600E
GNT35	GG	Meth	Meth 1	*BRAF-V600E, FGFR2-Q779A, CDKN2A/B Del, ATM-R2461C*
GNT48	GG	Meth	Meth 1	*BRAF*-V600E
GNT56	GG	Meth	Meth 1	*N/A*
GNT57	GG	Meth	Meth 1	*N/A*
GNT58	GG	Meth	Meth 1	*N/A*
GNT42	GG	Meth	Meth 1	*HIST1H3B*-M121T
GNT02	GNT NOS	Meth	Meth 2	*N/A*
GNT07	GNT NOS	Meth	Meth 2	*FGFR1* Duplication
GNT14	GNT NOS	Meth	Meth 2	*FGFR1* Duplication
GNT20	GNT NOS	Meth	Meth 2	*N/A*
GNT25	GNT NOS	Meth	Meth 2	*N/A*
GNT26	GNT NOS	Meth	Meth 2	*N/A*
GNT33	DNET	Meth	Meth 2	*FGFR1* Duplication
GNT34	DNET	Meth	Meth 2	*N/A*
GNT49	GG	Meth	Meth 2	*ARID1A*-158S, *ACVR1*-V435 Del
GNT31	DNET	Meth	Meth 2	*FGFR1*-L567E, *ATM*-R1039L
